# Application of 3D printed osteotomy guide plate-assisted total knee arthroplasty in treatment of valgus knee deformity

**DOI:** 10.1186/s13018-019-1349-9

**Published:** 2019-10-21

**Authors:** Zhimin Shen, Hong Wang, Yiqiang Duan, Jian Wang, Fengyan Wang

**Affiliations:** Department of Orthopedics, The Affiliated Hospital of Guizhou Medical University, No. 28, Guiyijie Road, Guiyang City, 550004 Guizhou Province China

**Keywords:** 3D printed osteotomy guide plate-assisted total knee arthroplasty, Valgus knee deformity, Clinical operation

## Abstract

**Introduction:**

To analyze the application of 3D printed osteotomy guide plate-assisted total knee arthroplasty (TKA) for valgus knee deformity.

**Methods:**

The clinical data of 20 patients with valgus knee deformity admitted to our hospital from April 2012 to April 2017 were collected and analyzed. According to the treatment method, these patients were divided into two groups: 3D printed osteotomy guide plate-assisted TKA (combined treatment group, *n* = 10) and TKA (treatment group, *n* = 10). The operation time, intraoperative bleeding volume, postoperative mean femorotibial angle (MFTA), and Knee Society Score (KSS) of the two groups were statistically analyzed.

**Results:**

Compared with the treatment group, the operation time was significantly shorter (*P* < 0.05), the intraoperative blood loss and postoperative MFTA were significantly decreased (*P* < 0.05), and the clinical and functional scores were significantly increased (*P* < 0.05) in the combined treatment group.

**Conclusion:**

3D printed osteotomy guide plate-assisted TKA for valgus knee deformity is more effective than TKA alone.

## Introduction

Total knee arthroplasty (TKA) is the most effective treatment of knee pain and dysfunction, which can reduce the pain of patients, restore the lower limb line, and reconstruct joint function [1]. It is also the gold standard procedure with excellent results for the treatment of advanced knee arthritis [2]. However, it is well known that valgus deformity with tibial plateau bone defect and dysplasia of the femoral condyle can lead to increased difficulty in TKA [3].

Valgus knee deformity is defined as a valgus angle equal to or greater than 10° and is observed in nearly 10% of patients undergoing TKA [4]. Excessive preoperative malalignment predisposes to a greater risk of failure [4]. The long-term results in valgus deformed knee were relatively inferior to varus deformation. One of the main reasons may be difficulty to acquire good soft tissue balance during surgery [2].

3D printing allows a surgeon to better visualize the anatomy in full 3D and digitally plan the osteotomy preoperatively based on CT images, resulting in improved accuracy of preoperative planning and surgical precision and decreased postoperative complication ratio [5–7]. It improves the accuracy of surgical resection by avoiding large segmental bone defects, improves the stability of knee joint after reconstruction, and improves the mechanical strength and stability of the prosthesis [8]. This technique is very precise and promising especially in patients requiring limb corrections in multiple planes [5]. At present, 3D printing technology has been used in the production of orthopedic experimental model, surgical auxiliary material printing, and printing of implant and joint surgery [9].

In this study, the operation time, intraoperative bleeding volume, mean femorotibial angle (MFTA), and Knee Society Score (KSS) between the application of 3D printed osteotomy guide plate-assisted TKA and artificial TKA for valgus knee deformity were compared.

## Materials and methods

### General information

The clinical data of 20 patients with valgus knee deformity admitted to our hospital from April 2012 to April 2017 were collected and analyzed. Inclusive criteria are as follows: all patients who were operated for the first time. Routine axial X-ray examination of the patella was performed before operation. Exclusive criteria are as follows: patients with surgical contraindications. According to the treatment method, these patients were divided into two groups: 3D printed osteotomy guide plate-assisted TKA (combined treatment group, *n* = 10) and TKA (treatment group, *n* = 10). There were 2 males and 8 females in the combined treatment group, aged 59–71 years, with an average of 60.3 ± 10.1 years old. According to their primary diseases, 6 patients had osteoarthritis and 4 patients had rheumatoid arthritis. According to the Keblish classification [10], 5 cases were mild, 4 cases were moderate, and 1 case was severe. There were 1 male and 9 females in the TKA treatment group, aged 60–71 years, mean 61.4 ± 10.2 years old. According to their primary diseases, 5 patients had osteoarthritis and 5 patients had rheumatoid arthritis. In terms of the Keblish classification, 4 cases were mild, 4 cases were moderate, and 2 cases were severe. The general data were comparable between the two groups (*P* > 0.05) (Table 1). This study was approved by the Ethics Committee of the authors’ hospital, and informed consent was taken prior to enrollment.

**Table 1:** Comparison of general data between the two groups of patients

Item	Classification	Combined treatment group (*n* = 10)	Treatment group (*n* = 10)	*t*/*χ*^2^	*P*
Gender	Male	2 (20.0)	1 (10.0)	2.71	> 0.05
	Female	8 (80.0)	9 (90.0)		
Age (year)		60.3 ± 10.1 (59–71)	61.4 ± 10.2 (60–71)	1.886	> 0.05
Primary diseases	Osteoarthritis	6 (60.0)	5 (50.0)	1.32	> 0.05
	Rheumatoid arthritis	4 (40.0)	5 (50.0)		
Keblish grading	Mild	5 (50.0)	4 (40.0)	2.77	> 0.05
	Moderate	4 (40.0)	4 (40.0)		
	Severe	1 (10.0)	2 (20.0)		

Data were presented as mean ± SD, *n* (percentage) or range. *n* number of cases

### Methods

#### Main instruments

The main instruments used were as shown in Table 2.

**Table 2:** Main instruments

Type of instrument	Conditions of use, materials, and characteristics	Source
Mimics 10.0	For processing images and creating 3D models. Can be used to import DICOM or raw image data, export 3D models for analysis, design or 3D printing, virtually plan a surgical procedure, create 3D models, and perform anatomical analysis. Uses 2D cross-sectional images such as from CT to construct 3D models, which can then be directly linked to RP, CAD, and surgical simulation.	Materialise, Leuven, Belgium
Creator Pro 3D printer	For creating 3D models. Equipped with versatile dual extruder, solid steel frame construction, and has a stable vertical movement.	FlashForge, Zhejiang, People’s Republic of China
64-row 128-slice volume CT (VCT)	For image acquisition. The scanning range includes the target knee joint. Scanning parameters: collimator 128 × 0.5 mm; tube voltage 120 kV, the tube current was automatically adjusted according to the patient’s body thickness; rotation time 0.5 s/r; layer thickness 3 mm, layer spacing 3 mm, reconstruction with soft tissue algorithm and bone algorithm, FOV = 512 × 512.	SOMATOM Definition AS, Siemens AG, Erlangen, Germany
Knee replacement prosthesis and surgical instruments	The knee replacement prosthesis included the femoral and tibial components.	DePuy Synthes, Raynham, MA, USA
CAD software (2012 version)	For modeling, measuring the digital model, and simulating surgery.	Autodesk Inc., San Rafael, CA, USA

### Treatment group

Patients in the treatment group received artificial TKA. The specific operation was as follows: Under general anesthesia, the patients were placed in a supine position, and an airbag tourniquet was used to pressurize the blood. The medial approach of the iliac crest was applied. First, routine femoral condyle and tibia plateau osteotomies were performed. Then, the posterior lateral joint capsule and lateral iliotibial band were completely loosened by using a pie-crusting technique. The standard was set to the outer compartment tension, and the rectangular spacer with appropriate thickness was placed in the knee joint extension gap. If the knee joint was still unstable under valgus stress after the rectangular straightening gap and the accurate lower limb force line were obtained, the starting point was set in the medial collateral ligament, and the osteotomy was upwardly slid, thereby tightening the medial collateral ligament.

### Combined treatment group

**Fig. 1:**
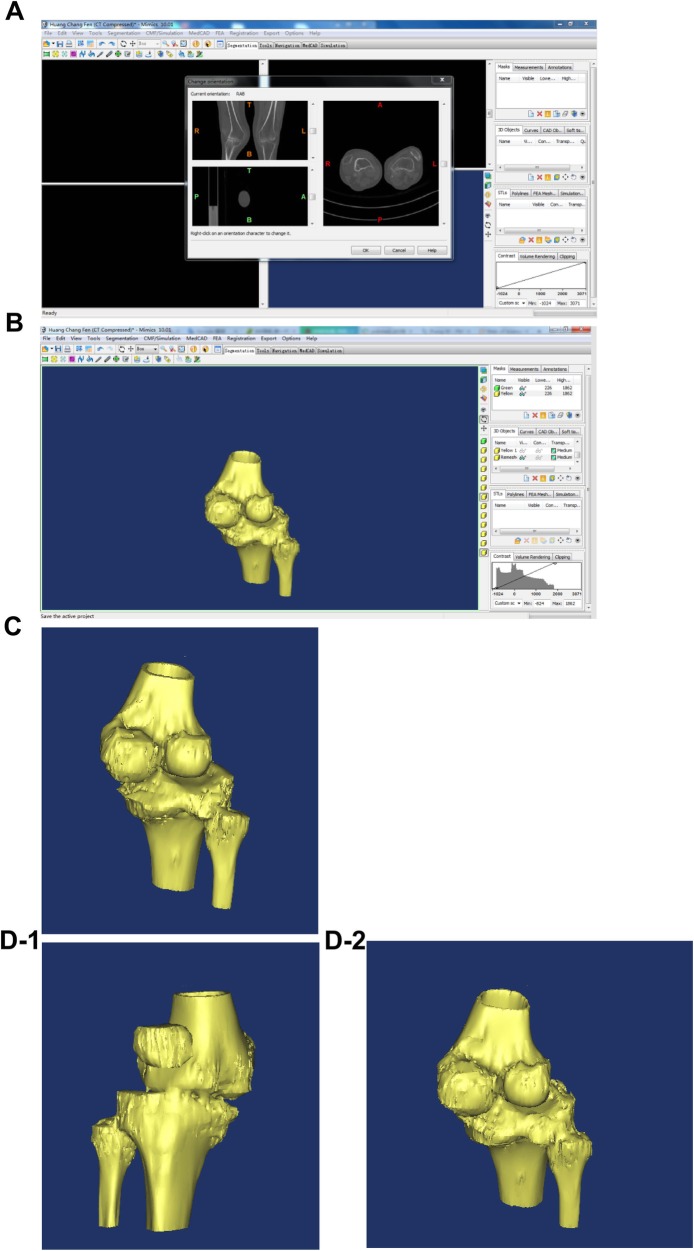
**A** Input data into Mimics software imaging. **B** Formation of 3D printed knee joint image in Mimics software. **C** Demonstration of 3D knee joint generated via Mimics software. Knee joint image generated **D-1** anterior-posterior view and **D-2** posterior view

**Fig. 2:**
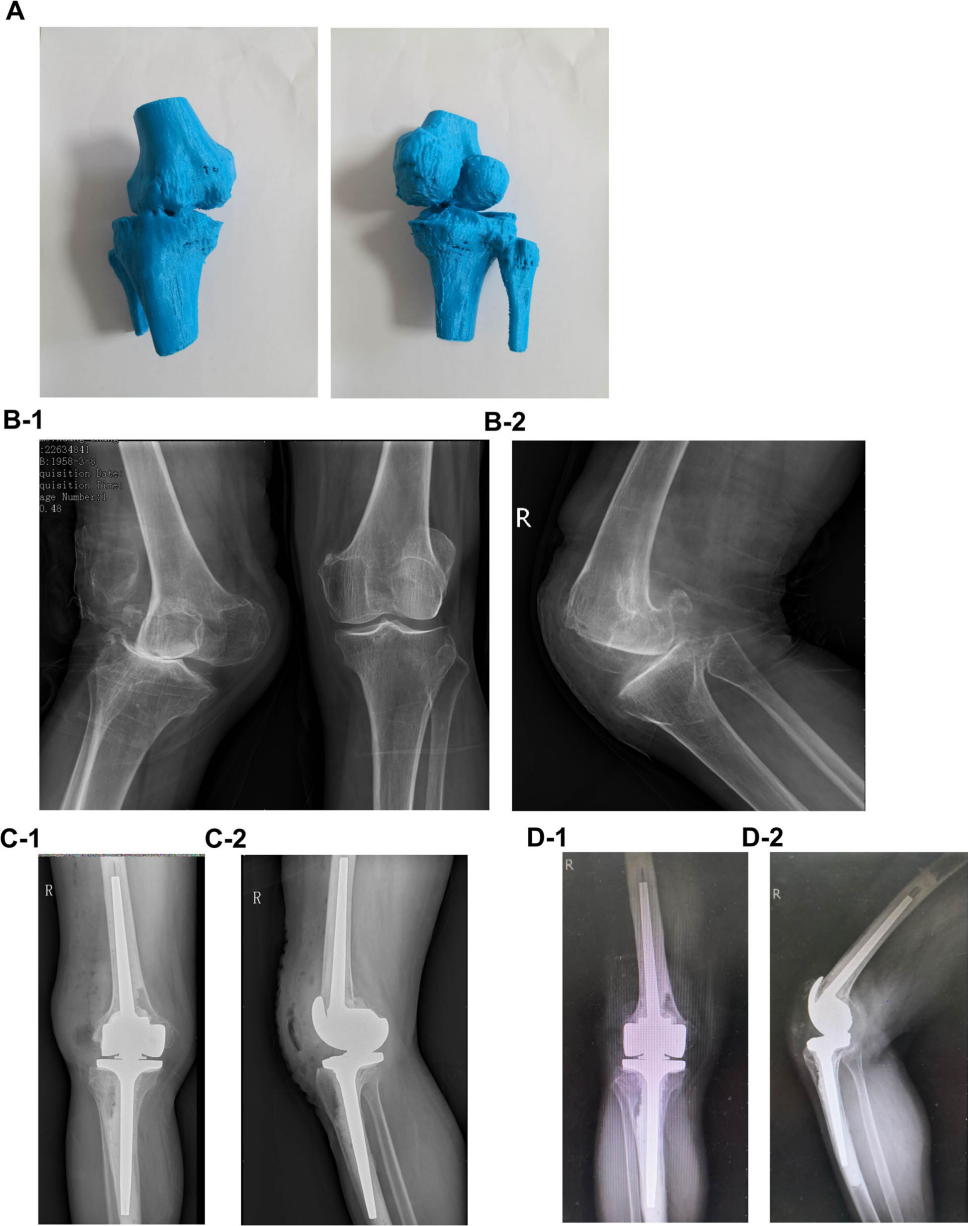
**A** 3D printing model. Preoperative anterior-posterior view (**B-1**) and lateral view (**B-2**). Postoperative anterior-posterior view (**C-1**) and lateral view (**C-2**). 1-year postoperative anterior-posterior view (**D-1**) and lateral view (**D-2**)

Patients in the combined treatment group received 3D printed osteotomy guide plate-assisted TKA. The procedure for artificial TKA was the same as above. The specific operation of the 3D printed osteotomy guide plate was as follows: CT image data from the patients of the femoral head to the ankle joint were collected before surgery to create the 3D printing model. In this process, the AutoCAD software was used to measure the digital model and simulate the surgery, a personalized osteotomy guide plate was then designed, and the patient’s knee anatomical model and osteotomy guide plate digital file entity were printed. Through the internal/lateral approach of the iliac crest, the knee joint was exposed, the joint effusion was absorbed, the osteophytes such as synovial tissue and meniscus were removed, and the lateral soft tissue was moderately loosed. The patient’s knee joint and 3D printed knee joint model were matched intraoperatively to eliminate loss of model accuracy caused by inaccurate image data or printing distortion. After determining the anatomy, the 3D femoral osteotomy guide plate was installed, ensuring the positioning module was attached to the anatomy of the knee joint. The femoral condyle was then osteotomied between the anterior and posterior oblique angles. Proximal tibia osteotomy was performed after the 3D tibial osteotomy guide plate was installed and was matched to the anatomical structure of the proximal tibia. The patient’s soft tissue balance was re-examined and moderately adjusted and was then pulse flushed. After the patient had installed the prosthesis and spacer, the force line was inspected to ensure that there was a good force line. Then, drainage was placed and the wound was sutured. Figure 1 shows some steps involved in 3D printed osteotomy guide plate-assisted TKA. Figure 2 shows the 3D printing model and pre- and postoperative radiographs of a patient with valgus knee deformity.

### Observation index

The operation time, intraoperative blood loss, and postoperative MFTA of the two groups were observed and measured. At the same time, the KSS was used to assess the clinical profile and function of the knee joint in the two groups before and after treatment. With the increase of score, the clinical profile and function of the knee joints were gradually improved [11].

### Statistical analysis

The data were analyzed by IBM SPSS statistical software (version 20.0) (IBM Corp., Armonk, NY, USA) and expressed as mean ± standard deviation (x̄ ± s). All values were analyzed using analysis of variance (ANOVA) and Newman-Keuls-Student’s *t* test. *P* < 0.05 was considered as statistically significant.

## Results

### Comparison of general data between the two groups

There was no significant difference in the general data between the two groups (*P* > 0.05, Table 1).

### Comparison of operation time, intraoperative blood loss, and postoperative MFTA between the two groups

The operation time, intraoperative blood loss, and postoperative MFTA were significantly lower in the combination group than the treatment group (*P* < 0.05, Table 3).

**Table 3:** Comparison of operation time, intraoperative blood loss, and postoperative MFTA between the two groups

Group	*n*	Operation time (min)	Intraoperative blood loss (ml)	Postoperative MFTA (°)
Combined treatment group	10	81.0 ± 10.0^*^	246.4 ± 43.3^*^	4.1 ± 0.1^*^
Treatment group	10	87.0 ± 10.2	293.0 ± 40.1	5.6 ± 0.7
*t*		3.182	4.303	2.776
*P*		< 0.05	< 0.05	< 0.05

Data were presented as mean ± SD. *n* number of cases

^*^P < 0.05, combined treatment group vs treatment group

### Comparison of KSS in the two groups before and after treatment

The clinical and functional scores of KSS in both groups after treatment were significantly higher than those before treatment (*P* < 0.05). There was no significant difference in the KSS clinical and functional scores between the two groups before treatment (*P >* 0.05). The clinical and functional scores of the patients after treatment in the combined treatment group were significantly higher than those in the treatment group (*P* < 0.05, Table 4).

**Table 4:** Comparison of KSS scores in the two groups before or after treatment

Group	*n*	Time	Clinical score	Functional score
Combined treatment group	10	Pretreatment	23.2 ± 3.1	29.0 ± 4.4
		Posttreatment	91.5 ± 10.1^#*^	90.3 ± 10.8^#*^
Treatment group	10	Pretreatment	23.0 ± 5.1	27.3 ± 4.1
		Posttreatment	77.3 ± 10.3^#^	77.0 ± 10.6^#^

Data were presented as mean ± SD. *n* number of cases

^#^*P* < 0.05, posttreatment vs pretreatment

^*^P < 0.05, combined treatment group vs treatment group

## Discussion

The desired thickness and angle of osteotomy which is determined by preoperative X-ray, intraoperative extramedullary positioning devices, and clinician’s experience in routine TKA is easy to produce certain deviations, resulting in surgical failure [12]. Studies have shown that the accuracy of the osteotomy angle determined by conventional surgical instruments is only 75% compared with actual anatomy [13–17]. The coincidence rate will have a larger gap if the patient has severe cartilage loss and knee deformity. The 3D printed osteotomy guide plate takes full advantage of the two core technologies of rapid prototyping (RP) and computer-aided design (CAD). The CAD software can build a 3D model based on the patient’s CT data. Meanwhile, it can measure, cut, and match anatomical models of patients [18]. In addition, CAD software can accurately measure anatomical structures before TKA and accurately reconstruct lower limb force lines [19]. It can be applied to precise digital orthopedics operation of bone and joint deformity [20], while increased precision are good indicators of how 3D guides reduce potential human error [21]. Furthermore, the 3D printed osteotomy guide plate can effectively assist in TKA operation [22].

To develop a 3D model based on the patient’s CT data, first, the 64-row 128-slice VCT was used for scanning to obtain the thin-slice CT data. The relevant parameters were set according to the actual situation of each patient, and the obtained data were saved in Digital Imaging and Communications in Medicine (DICOM) format and reserved for use. Second, data transmission was conducted. The surgical transmission mode was selected to transmit the acquired data to Mimics 10.0 software. The “Import Image” command, import image and data, and lossless compression mode were selected, and according to the left and right orientation of the image and sagittal image, the anterior-posterior position was determined to complete the data import. Third, 2D image production was performed. In this study, bone window and soft tissue window scanning was used for CT data, and 3D reconstruction was performed. Combined with 2D images of the transverse plane, sagittal plane, and coronal plane, an appropriate threshold range was selected to separate the relevant tissues of the obtained images and generate the 2D bone tissue contour, which was the original mask. Fourth is the separation and editing of mask. On the basis of the original mask, the function of “2D regional growth” was used to select the bone which connects with the main body, and combined with the transverse, sagittal, and coronal planes, the corresponding knee joint was selected and the 3D model of the knee joint mask was calculated. Fifth is the reconstructing of high simulation 3D model. According to the reconstruction requirement of the model, the relevant parameters were set and “High” was selected to improve the quality of the model. The other parameters were default values, which made the model more intuitive and realistic, and had higher simulation and visualization. Rotation, translation, enlargement, and reduction of the model were performed in the interface to understand the knee joint condition.

In the acute treatment of genu valgus, 3D printed osteotomy guide plate-assisted TKA does not require the conventional application of intramedullary and intramedullary positioning system, measurement of prosthesis size, and rotation of femur to measure the module. It is a relatively simple surgical operation and has a relatively short surgical time [23, 24]. The potential clinical advantage of time reduction includes the association of lower infection rates [25]. Since the medullary cavity opening is not required during the operation, the intraoperative and postoperative blood loss and the risk of fat embolism are reduced [26].

## Conclusion

The present study showed that the operative time, intraoperative blood loss, and postoperative MFTA of patients in the combined treatment group were significantly lower than those in the treatment group, while the clinical and functional scores were significantly higher than those in the treatment group. These results indicate that the clinical effect of 3D printed osteotomy guide plate-assisted TKA in the treatment of valgus knee deformity is better than that of TKA alone, which is worthy of promotion.

## Data Availability

The datasets used and analyzed during the current study are available from the corresponding author on reasonable request.

## Abbreviations

2D: Two-dimensional
3D: Three-dimensional
CAD: Computer-aided design
DICOM: Digital Imaging and Communications in Medicine
KSS: Knee Society Score
MFTA: Mean femorotibial angle
Mimics: Materialise’s Interactive Medical Image Control System
RP: Rapid prototyping
TKA: Total knee arthroplasty
VCT: Volumetric computed tomography

## References

[1] Bistolfi A, Massazza G, Rosso F, Deledda D, Gaito V, Lagalla F (2011). Cemented fixed-bearing PFC total knee arthroplasty: survival and failure analysis at 12-17 years. J Orthop Traumatol..

[2] Nikolopoulos D, Michos I, Safos G, Safos P (2015). Current surgical strategies for total arthroplasty in valgus knee. World J Orthop..

[3] Helmy Naeder, Dao Trong Mai Lan, Kühnel Stefanie P. (2014). Accuracy of Patient Specific Cutting Blocks in Total Knee Arthroplasty. BioMed Research International.

[4] Rossi R, Rosso F, Cottino U, Dettoni F, Bonasia DE, Bruzzone M (2014). Total knee arthroplasty in the valgus knee. Int Orthop..

[5] Hoekstra H, Rosseels W, Sermon A, Nijs S (2016). Corrective limb osteotomy using patient specific 3D-printed guides: a technical note. Injury..

[6] Victor J, Premanathan A (2013). Virtual 3D planning and patient specific surgical guides for osteotomies around the knee: a feasibility and proof-of-concept study. Bone Joint J..

[7] Rengier F, Mehndiratte A, von Tengg-Kobligk H, Zechmann CM, Unterhinninghofen R, Kauczor HU (2010). 3D printing based on imaging data: review of medical applications. Int J Comput Assist Radiol Surg..

[8] Wang F, Zhu J, Peng X, Su J (2017). The application of 3D printed surgical guides in resection and reconstruction of malignant bone tumor. Oncol Lett..

[9] Ma L, Zhou Y, Zhu Y, Lin Z, Wang Y, Zhang Y, Xia H, Mao C (2016). 3D-printed guiding templates for improved osteosarcoma resection. Sci Rep..

[10] Keblish PA (1991). The lateral approach to the valgus knee. Surgical technique and analysis of 53 cases with over two-year follow-up evaluation. Clin Orthop Relat Res..

[11] Desseaux A, Graf P, Dubrana F, Marino R, Clavé A (2016). Radiographic outcomes in the coronal plane with iASSIST^TM^ versus optical navigation for total knee arthroplasty: a preliminary case. Orthop Traumatol Surg Res..

[12] Schiraldi M, Bonzanini G, Chirillo D, de Tullio V (2016). Mechanical and kinematic alignment in total knee arthroplasty. Ann Transl Med..

[13] Park A, Nam D, Friedman MV, Duncan ST, Hillen TJ, Barrack RL (2015). Inter-observer precision and physiologic variability of MRI land-marks used to determine rotational alignment in conventional and patient-specific TKA. J Arthroplasty..

[14] Matsuda S, Kawahara S, Okazaki K, Tashiro Y, Iwamoto Y (2013). Postoperative alignment and ROM affect patient satisfaction after TKA. Clin Orthop Relat Res..

[15] Ma B, Kunz M, Gammon B, Ellis RE, Pichora DR (2014). A laboratory comparison of computer navigation and individualized guides for distal radius osteotomy. Int J Comput Assist Radiol Surg..

[16] Qiao F, Li D, Jin Z, Gao Y, Zhou T, He J, Cheng L (2015). Application of 3D printed customized external fixator in fracture reduction. Injury..

[17] Bäthis H, Perlick L, Tingart M, Lüring C, Zurakowski D, Grifka J (2004). Alignment in total knee arthroplasty. A comparison of computer-assisted surgery with the conventional technique. J Bone Joint Surg Br..

[18] Renson L, Poilvache P, Van den Wyngaert H (2014). Improved alignment and operating room efficiency with patient-specific instrumentation for TKA. Knee..

[19] Olszewski R (2013). Three-dimensional rapid prototyping models in craniomaxillofacial surgery: systematic review and new clinical applications. P Belg Roy Acad Med..

[20] Ding HW, Shen JJ, Tu Q, Wang YJ, Zhang DH, Wang H (2011). Application of computer aided technique in osteoarthrosis. J Clin Rehabilit Tissue Eng Res..

[21] Arnal-Burró J, Pérez-Mañanes R, Gallo-Del-Valle E, Igualada-Blazquez C, Cuervas-Mons M, Vaquero-Martín J (2017). Three dimensional-printed patient-specific cutting guides for femoral varization osteotomy: do it yourself. Knee..

[22] Shen C, Tang ZH, Hu JZ, Zou GY, Xiao RC, Yan DX (2015). Patient-specific instrumentation does not improve accuracy in total knee arthroplasty. Orthopedics..

[23] Lionberger DR, Crocker CL, Chen V (2014). Patient specific instrumentation. J Arthroplasty..

[24] Conteduca F, Iorio R, Mazza D, Caperna L, Bolle G, Argento G (2013). Evaluation of the accuracy of a patient-specific instrumentation by navigation. Knee Surg Sports Traumatol Arthrosc..

[25] Thanni LO, Aigoro NO (2004). Surgical site infection complicating internal fixation of fractures: incidence and risk factors. J Natl Med Assoc..

[26] Issa K, Rifai A, McGrath MS, Callaghan JJ, Wright C, Malkani AL (2013). Reliability of templating with patient-specific instrumentation in total knee arthroplasty. J Knee Surg..

